# A sexual rehabilitation intervention for women with gynaecological cancer receiving radiotherapy (SPARC study): design of a multicentre randomized controlled trial

**DOI:** 10.1186/s12885-021-08991-2

**Published:** 2021-12-04

**Authors:** Isabelle Suvaal, Susanna B. Hummel, Jan-Willem M. Mens, Helena C. van Doorn, Wilbert B. van den Hout, Carien L. Creutzberg, Moniek M. ter Kuile

**Affiliations:** 1 Department of Gynaecology, Leiden University Medical Center, Zone K6-T, PO Box 9600, 2300 RC Leiden, the Netherlands; 2 Department of Radiotherapy, Erasmus Medical Center Cancer Institute, Rotterdam, the Netherlands; 3 Department of Gynaecology, Erasmus Medical Center Cancer Institute, Rotterdam, the Netherlands; 4 Department of Biomedical Data Sciences, Leiden University Medical Center, Leiden, the Netherlands; 5 Department of Radiation Oncology, Leiden University Medical Center, Leiden, the Netherlands

**Keywords:** Health-related quality of life, Sexual functioning, Rehabilitation intervention, Gynaecological cancer

## Abstract

**Background:**

Sexual problems are frequently reported after treatment with radiotherapy (RT) for gynaecological cancer (GC), in particular after combined external beam radiotherapy and brachytherapy (EBRT+BT). Studies demonstrate that psychosexual support should include cognitive behavioural interventions and involvement of the patient’s partner, if available. Therefore, we developed a nurse-led sexual rehabilitation intervention, including these key components. The intervention was previously pilot-tested and results demonstrated that this intervention improves women’s sexual functioning and increases dilator compliance. The objective of the current study is to investigate the (cost-)effectiveness of the intervention compared to optimal care as usual (CAU). We expect that women who receive the intervention will report a statistically significant greater improvement in sexual functioning and – for women who receive EBRT+BT – higher compliance with dilator use, from baseline to 12 months post-RT than women who receive optimal care as usual (CAU).

**Methods/design:**

The intervention is evaluated in the SPARC (Sexual rehabilitation Programme After Radiotherapy for gynaecological Cancer) study, a multicentre, randomized controlled trial (RCT). The primary endpoint is sexual functioning. Secondary outcomes include body image, fear of sexual activity, sexual-, treatment-related- and psychological distress, health-related quality of life and relationship satisfaction. A cost-effectiveness analysis (CEA) will be conducted in which the costs of the intervention will be related to shifts in other health care costs and the impact on patient outcome. The study sample will consist of 220 women with GC treated with RT in specialized GC treatment centres (N = 10). Participants are randomized to either the intervention- or CAU control group (1:1), and within each centre stratified by type of radiotherapy (EBRT+BT vs. EBRT only) and having a partner (yes/no). All women complete questionnaires at baseline (T1) and at 1, 3, 6, and 12 months post-RT (T2, T3, T4 and T5, respectively).

**Discussion:**

There is a need to improve sexual functioning after RT for GC. This RCT will provide evidence about the (cost-)effectiveness of a nurse-led sexual rehabilitation intervention. If proven effective, the intervention will be a much needed addition to care offered to GC survivors and will result in improved quality of life.

**Trial registration:**

ClinicalTrials.gov, NCT03611517. Registered 2 August 2018.

## Background

In the Netherlands, more than 4500 women are diagnosed with gynaecological cancers (GC) annually [1]. Approximately one-third of GC patients, especially those with cervical, uterine and vaginal cancers, receive radiotherapy (RT) as primary or post-surgical treatment; most often external beam radiotherapy (EBRT) with or without brachytherapy (BT). Sexual problems, such as dyspareunia, vaginal dryness and a decrease of sexual satisfaction and desire are frequently reported after treatment with RT for GC [2–9]. The negative effects of RT, and in particular of the combination of EBRT with BT (EBRT+BT), on sexual functioning are due to vaginal changes such as fibrosis with vaginal shortening and tightening, mucosal atrophy, and reduced flexibility and decreased lubrication of the upper vagina [10]. EBRT+BT is the standard combination for primary treatment of more advanced stages of cervical, vaginal and endometrial cancer, while postoperative BT is only added in case of involved or tight vaginal margins.

To prevent or reduce vaginal shortening and tightening during the period of fibrosis formation after EBRT+BT, it is generally recommended to use vaginal dilators for a period of 9-12 months after completion of treatment [11]. Such use may prevent or minimise vaginal stenosis with the purpose to maintain the option of vaginal penetration in the long term [11, 12]. Despite the proposed benefits of regular dilator use, most GC patients (75%) fail to use dilators regularly even after counselling and instructions for use [13–16]. Frequently reported barriers to dilator use are difficulties with planning, lack of time or privacy, forgetting, and other problems in the recovery phase such as fatigue and worry [17]. To increase compliance, it is important to provide sufficient patient information tailored to the woman’s need, clear instructions and psycho-education regarding dilator use [18]. In addition, the process of recovery from cancer diagnosis and treatment and the associated physical and psychological problems is already demanding. A broader view on rehabilitation, counselling and support would be needed to help multi-dimensional recovery and increase compliance with regular dilator use.

A recent study demonstrated that GC patients treated with RT benefit from a psychosexual rehabilitation information booklet [19]. Women reported more knowledge regarding physical and psychosexual side-effects and rehabilitation options in the first 6 months post-RT than women wo received standard information materials. However, the psychosexual rehabilitation booklet did not increase compliance with dilator use. In addition to psycho-education, two studies demonstrated that a psychoeducational group intervention, including a focus on motivation to engage in regular dilator use, increased dilator compliance [15, 20]. Indicating that GC survivors could benefit from additional professional support targeting dilator use. However, such an intervention targeting dilator use only did not affect the psychosexual consequences of treatment of GC, such as decreased sexual desire, dyspareunia, diminished body image, and relationship dissatisfaction [15]. Therefore, psychosexual rehabilitation interventions should focus on preventing and reducing RT-induced vaginal changes, as well as on increasing psychosexual and relationship satisfaction [21].

Only few studies have evaluated psychosexual rehabilitation interventions – which used cognitive behavioural techniques, psycho-education and counselling – for GC survivors [22–26]. Results demonstrated that women who received the psychosexual rehabilitation interventions experienced better sexual functioning, less sexual distress [23] and a decrease in sexual problems [22]. Only one of the studies actively involved the partner in the intervention and demonstrated that a couple-coping training improved sexual relationship satisfaction to a greater extent than medical information education or patient-coping training only [26].

It can be concluded that psychosexual support after treatment for GC should include motivational issues and psychosexual interventions to increase dilator compliance and improve sexual functioning. Furthermore, involvement of the partner is preferred. There is a need for a condensed, practical and (cost-)effective sexual rehabilitation intervention, consisting of psycho-education combined with elements of psychosexual-based cognitive behavioural therapy for GC patients and their partners after RT [27–31]. Therefore, based on the results of our previous studies [17, 18, 21], we developed a nurse-led sexual rehabilitation intervention to support sexual improvement and vaginal dilator use after RT. The intervention has multiple aims: motivating women, giving tailored advice, strengthening self-management, promoting couples’ mutual coping and support processes and, for women who received EBRT+BT, providing information and coaching on use of vaginal dilators on a regular basis. The results of our pilot study regarding the feasibility of the intervention demonstrated that this intervention improved women’s sexual functioning and that it supported them in their dilator use [32]. Furthermore, the nurses who were trained and supervised to guide the intervention felt capable to support the women.

In this manuscript we present the design of the SPARC (Sexual rehabilitation Programme After Radiotherapy for gynaecological Cancer) study, a multicentre randomized controlled trial (RCT) which evaluates the (cost-)effectiveness of the nurse-led sexual rehabilitation intervention in improving sexual functioning and dilator use of GC patients after RT. Participants are randomized to either the intervention- or optimal care as usual (CAU) control group (1:1). We expect that women who receive the intervention will report a statistically significant greater improvement in sexual functioning and – for women who receive EBRT+BT – higher compliance with dilator use, from baseline to 12 months post-RT than women who receive CAU.

## Methods

**Fig. 1:**
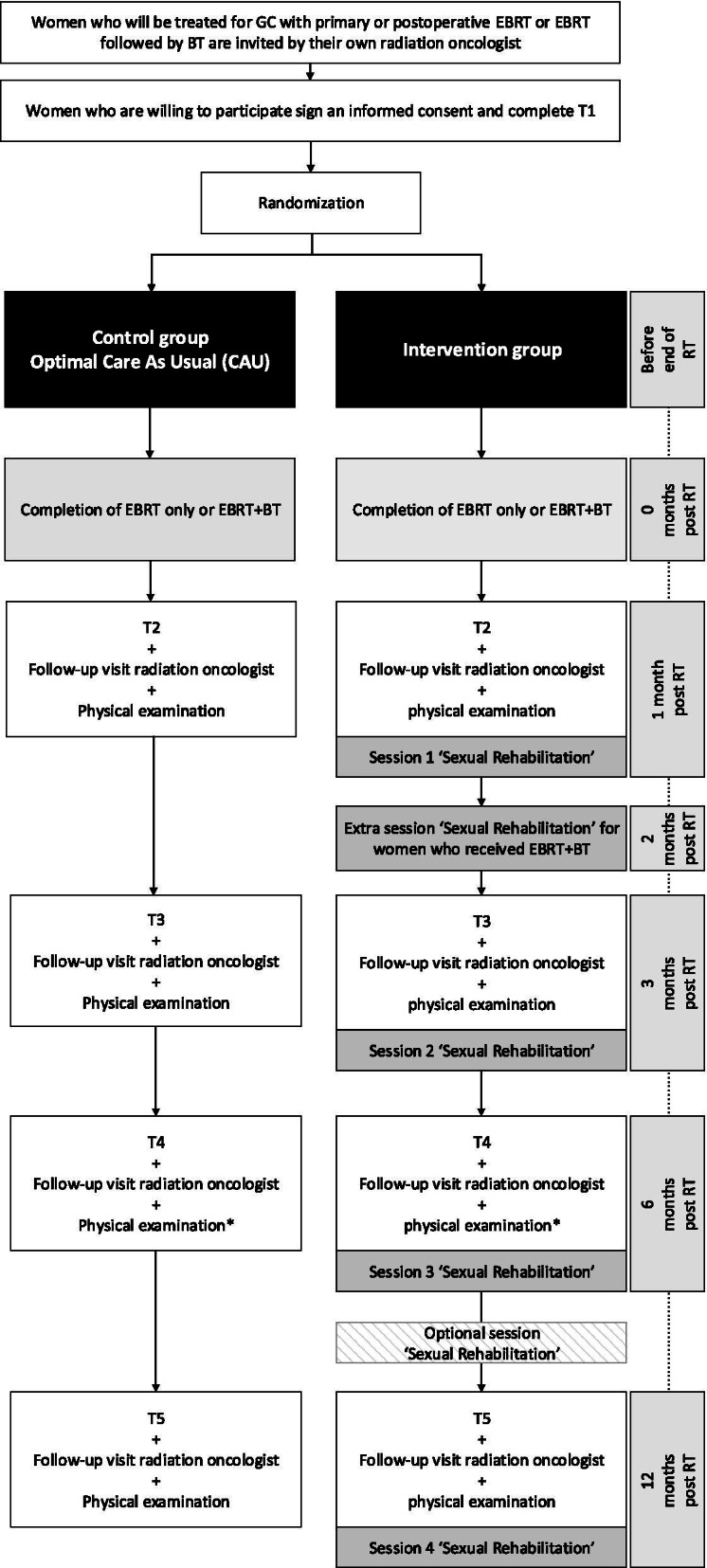
Overview of study procedures. *The physical examination at the 6 months follow-up visit might also be conducted by the gynaecologist (depending on the centre). BT = Brachytherapy; EBRT = External Beam Radiotherapy; GC = Gynaecological cancer; RT = Radiotherapy; T1 = Self-report baseline questionnaire, T2-5 = Self-report follow-up questionnaires

The study design and main procedures of the RCT are displayed in Fig. 1.

### Ethical issues, safety aspects and (medical) complications

The SPARC study has been approved by the Medical Ethical Committee Leiden-Den Haag-Delft (number NL62767.058.17) and by the institutional review boards of the 10 participating GC centres (the participating GC centres are included in the Acknowledgements section). Recruitment and data collection started in August, 2018 and is still ongoing at the date of publication of this manuscript. This study is monitored on data and safety by an independent certificated study monitor. All study-related adverse events, i.e. dilator-use-related, will be recorded an reported as an adverse event. Events related to the undergone cancer treatments are not considered study-related events.

### Study Sample

The study sample will consist of 220 women diagnosed with cervical, vaginal or endometrial cancer and who will receive primary or postoperative EBRT+BT (BT boost by intra-uterine and/or vaginal brachytherapy) or postoperative EBRT alone. This also includes curative treatment with EBRT+BT for local relapse after previous surgery. Participants are recruited in specialized GC treatment centres in the Netherlands. Women are eligible for study participation if they (1) are 18 years or older, (2) receive treatment with RT for GC as specified above and (3) wish to retain their sexual activity in the short- or long term. Women are excluded from study participation if they (1) are unavailable for follow-up, (2) have insufficient command of the Dutch language or (3) have a major affective disorder, psychotic disorder, substance abuse disorder or posttraumatic stress disorder resulting from abuse in the pelvic floor area and/or genitals. To ensure appropriate treatment of these women with more severe psychological and/or psychiatric problems, they are referred to a specialized psychologist/sexologist connected to the own GC centre or are advised to consult their general practitioner to be referred to a specialized psychologist/sexologist or psychiatrist.

### Recruitment and randomization

The treating radiation oncologists screen potential participants with regard to the inclusion and exclusion criteria. Eligible women are informed about the background, rationale and specifics of the study protocol. Women who want to participate in the study provide written informed consent and complete a paper-and-pencil baseline questionnaire before randomization. After inclusion, participants are assigned a unique study identifier by the local data manager, which will be filled out on all questionnaires and used in the data files. Inclusion and randomization take place before the completion of RT. Participants are randomized to either the intervention- or the optimal CAU control group (1:1), and within each centre stratified by type of radiotherapy (EBRT+BT versus EBRT only) and having a partner (yes/no). The randomization is block stratified with varying block sizes, and is performed by the local data manager, using a secured web-based data-management system.

### Study groups

One month after completion of RT, women in both study groups receive an information booklet. The information booklets, which contain specific information to improve knowledge and coping strategies regarding sexual rehabilitation after either EBRT+BT or EBRT only, are based on the booklet developed in the pilot study [32]. The booklets are provided in both a printed and online version. Women in both study groups who received EBRT+BT are given a dilator set and are advised to start vaginal dilation for 1 to 3 min, 2 to 3 times a week around 4 weeks after RT when the vagina is sufficiently healed [11, 18].

#### Training of the nurses

The intervention is conducted by oncology nurses who have completed a 50-hour study-specific training in sexology and simple cognitive behavioural interventions and the treatment protocol itself. The nurses are supervised by an experienced sexologist once per month. Every six months, the oncology nurses and their supervisors attend an additional day of training that is focused on a specific theme that is relevant for the study (i.e. vaginal stenosis and dilator use, emotional reactions after loss of participants due to cancer recurrence, and the partner relationship).

#### Intervention group: the nurse-led sexual rehabilitation intervention

The intervention consists of four one-hour face-to-face sessions at 1, 3, 6, and 12 months post-RT. These sessions are planned synchronously with women’s radiation oncologist follow-up visits. An additional session is scheduled for women who received EBRT+BT at 2 months, during which potential barriers and problems with dilator use are discussed. Furthermore, if preferred, an extra follow-up session/telephone consultation of 30 min can be scheduled between 6 and 12 months after RT. If a woman is in a relationship, the partner is invited to accompany her in the sessions.

**Fig. 2:**
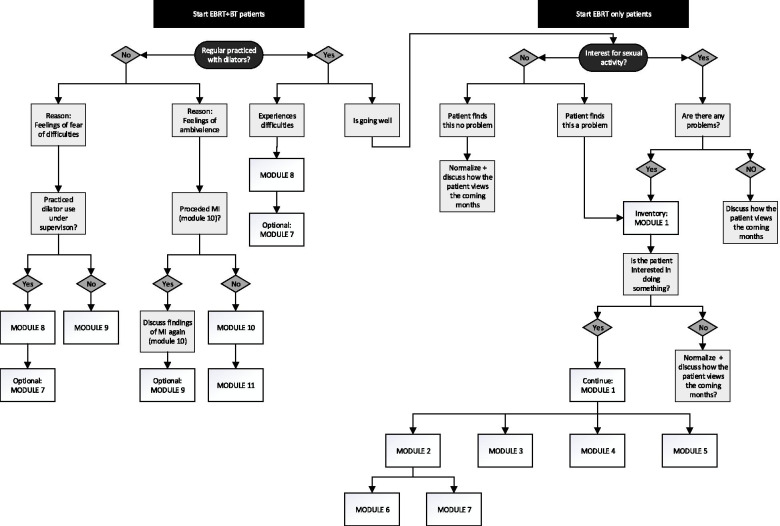
Decision Tree intervention modules. BT = Brachytherapy; EBRT = External Beam Radiotherapy; MI = Motivational Interviewing [35]; Module 1 = Brief sexual history; Module 2 = Pain during intercourse; Module 3 = Vaginal dryness and health; Module 4 = Alternatives for intercourse; Module 5 = The partner and possible sexual problems; Module 6 = Gradual exposure towards sexual intercourse; Module 7 = Pelvic floor exercise; Module 8 = Difficulties with dilator use at home; Module 9 = Using dilators under supervision at the outpatient clinic; Module 10 = Exploring and resolving ambivalence with regard to dilator use; Module 11 = Petroleum jelly (Vaseline) tampon

The sexual rehabilitation intervention consists of 11 modules. A description of the content of the modules is provided in Table 1. The modules include topics such as education regarding the specific cancer diagnosis and treatment, education on the importance of regular use of vaginal dilators (if applicable), discussing potential experienced barriers to dilator use (if applicable) and lubricant use, fear of penetration with dilators (if applicable) and fear of resuming sexual activity after cancer, promoting couples’ mutual coping and support processes and addressing sexual and body image concerns. The content of the intervention is a personalized programme tailored to the participant-specific psychological, relational and somatic factors. During a session, the oncology nurse selects the specific module(s) that fits the woman’s (and her partner’s) needs. The decision tree for module selection is provided in Fig. 2.

**Table 1:** Description of the sexual rehabilitation intervention modules

**Module 1: Brief sexual history**	This module describes how the nurse can question the patient in-depth about sexual problems on various domains of sexual functioning, including sexual interest/arousal, orgasm, pain and sexual satisfaction. It also covers psycho-education about sexuality and the sexual response curve [33] and provides information about frequently occurring sexual problems and solutions.
**Module 2: Pain during intercourse**	This module includes practical guidelines that the nurse can provide regarding pain during intercourse after radiotherapy-, with referrals to module 3, 4, 6 and 7, and explains how to provide psycho-education about the circular model of dyspareunia [34], which is based on a cognitive behavioural framework.
**Module 3: Vaginal dryness and health**	This module provides the nurse with instructions on how to give advice with regard to treatment of vaginal dryness, pain or irritation. It also includes information regarding vaginal health, such as the use of vaginal creams, avoidance of scratching in response to irritated skin or avoidance of washing with soap.
**Module 4: Alternatives for intercourse**	The exercise in this module helps the woman and her partner (if available) to explore and discuss non-penetrative alternatives for sexual intercourse.
**Module 5: The partner and possible sexual problems**	This module can be consulted by the nurse when partners experience temporary sexual problems, such as erectile dysfunction during intercourse. The module also includes a reference to module 1.
**Module 6: Gradual exposure towards sexual intercourse**	The aim of the steps in this module, which are based on a cognitive behavioural gradual exposure therapy for Genito-Pelvic/Penetration Disorder [29], is to learn the woman and her partner how to re-engage in sexual intercourse. The steps include: touching of the vaginal opening with the erect penis without penetration, stepwise vaginal insertion of the erect penis without moving, and vaginal insertion of the erect penis with moving.
**Module 7: Pelvic floor exercise**	This module includes several pelvic floor relaxation exercises for women who experience tension in the pelvic floor muscles.
**Module 8: Difficulties with dilator use at home**	This module is suitable for women who experience problems with dilator use and who already practiced under supervision of a nurse (see module 9) or for women who do not want to practice under supervision. This module provides the nurse with instructions on how to give specific advice on how to overcome experienced difficulties, after first exploring the problems during dilator use (e.g. pain/discharge, loss of blood or difficulties with inserting the dilator).
**Module 9: Using dilators under supervision at the outpatient clinic**	This module focuses on women who experience fear with regard to dilator use or who experience difficulties when using vaginal dilators, due to for example tension of the pelvic floor. The nurse-led session is based on therapist-aided exposure therapy for Genito-Pelvic/Penetration Disorder [29]. The goal is to reduce fear of dilator use by using a stepwise exposure session in which the woman - who performs the vaginal dilation by herself - is facilitated by the nurse. During the session, tips are given with regard to a correct and more comfortable use of the dilators. Furthermore, the nurse helps to evaluate and articulate any unhelpful cognitions about what could (or could not) occur during dilator use. In these instances, the exposure is used as a behavioural experiment, to test the tenability of these cognitions. The module also includes advice on how to handle problems that might occur during practicing at home.
**Module 10: Exploring and resolving ambivalence with regard to dilator use**	The aim of the exercise in this module is to motivate the woman for dilator use, by acknowledging, exploring and resolving ambivalent feelings towards dilator use by motivational interviewing technique [35]. By exploring pros and cons of both dilator use and no dilator use, the woman can be supported in making an informed choice about dilator use. If she decides to use dilators, problems with dilator use are discussed in more detail and how to overcome them. If a woman decides not to use dilators, tampons covered in petroleum jelly (Vaseline) are recommended and guidelines on how to use these are provided to the woman (see module 11).
**Module 11: Petroleum jelly (Vaseline) tampons**	This module follows module 10, when a woman decides not to use dilators. The module covers guidelines on how to use tampons covered in petroleum jelly (Vaseline).

All sessions will be audio-taped allowing a direct check on the oncology nurses’ adherence and competency. A random check of 30% of audio-taped sessions on adherence of the treatment protocol will be performed by two independent researchers. Furthermore, these audio-taped treatment sessions will be used for supervision purposes.

#### Control group: Optimal Care As Usual

All participating women are offered an information session, information booklet and a dilator set (if applicable) post-RT free of charge. Hereby, we optimized the CAU control group in our current study. Although CAU cannot be completely standardized, as the procedure is dependent on the local standard practice, it will not involve a structured, tailored nurse-led sexual rehabilitation intervention.

### Data collection

Participants are requested to complete questionnaires prior to randomization (T1, baseline) and at 1, 3, 6, and 12 months post-RT (T2, T3, T4 and T5, respectively, see Fig. 1). The T1 questionnaire is completed on paper. The T2-T5 study questionnaires can be completed either online (a link is sent by email) or on paper, depending on the preference of the participant. An online reminder is sent to participants who do not complete and return the questionnaire within one week. If a participant does not complete the questionnaire in the week following the reminder, she is contacted by telephone. Vaginal symptoms, assessed by standardized clinical examination during a physical examination, are synchronously collected at the timepoints of the T1-T5 questionnaires through Case Report Forms (CRFs) and medical records. For women who withdraw early from study participation, data collected until the moment of withdrawal will be available for analysis.

### Measures

#### Sociodemographic and clinical data

Sociodemographic data are obtained via the T1 questionnaire and include age, relational status, living situation, having children, education, and work status. Clinical data are collected from medical records and through CRFs at T1 and include date of gynaecological cancer diagnosis, type of gynaecological cancer (cervical/endometrial/vaginal) and characteristics (histological type, Fédération Internationale de Gynécologie et d’Obstétrique (FIGO) stadium, lymph node metastases), treatment(s) received (surgery, chemotherapy, type of radiotherapy, hypothermia), height and weight, and smoking. In addition, World Health Organisation (WHO) performance status, menopausal status and medication use (including hormonal replacement therapy) are collected from medical records and through CRFs at T1-T5.

#### Outcome measures

Detailed descriptions of the outcome measures are provided in Table 2. The primary outcome measure is a standardized patient-reported outcome measure (PROM) assessing sexual functioning (Female Sexual Function Index (FSFI) [36]). The secondary outcome measures include PROMs assessing vaginal symptoms and body image concerns (European Organization for Research and Treatment of Cancer Quality of Life Questionnaire-Gynaecological Cancer Module (EORTC QLQ-CX24) [37]), fear of coital and non-coital sexual activity (Fear of Sexuality Questionnaire (FSQ) [38]), sexual distress (Female Sexual Distress Scale (FSDS) [39]), treatment-related distress (Impact of Event Scale (IES) [40]), generic health-related quality of life (European Organization for Research and Treatment of Cancer Quality of Life Questionnaire Core 30 (EORTC QLQ-C30) [41]), urological and gastrointestinal symptoms and sexual interest (European Organization for Research and Treatment of Cancer Quality of Life Questionnaire-Endometrial Cancer Module (EORTC QLQ-EN24) [42]), psychological distress (Hospital Anxiety and Depression Scale (HADS) [43]), and relationship satisfaction (Maudsley Marital Questionnaire (MMQ) [44]). To minimize respondent burden, the T1-questionnaire includes only the FSFI [36] and the FSDS [39].

**Table 2:** Study outcome measures and corresponding questionnaires

Variable	Questionnaire	Details
**Primary Outcome**		
Sexual functioning	FSFI [36, 45]	• Assesses overall sexual functioning • 19 items; 5- and 6-point Likert scales • Subscales: sexual desire; arousal; lubrication; orgasm; satisfaction; pain • Total score*: 2-36/Subscale scores*: desire 1.2-6; arousal 0-6; lubrication 0-6; orgasm 0-6; satisfaction 0-6. Higher score indicates better overall sexual functioning. A subscale score of 0 indicates no sexual activity • Time frame: past 4 weeks • Cronbach’s alpha in a gynaecologic cancer survivors group: total score: α = 0.94; subscale scores: 0.85 ≤ α ≤ 0.94 [46] • We added 4 items (6- and 7-point Likert scales) to assess the average frequency and amount of pleasure experienced during sexual activity without sexual intercourse and sexual activity with sexual intercourse • As our study sample consists of partnered as well as unpartnered women, we added an answer option ‘not applicable, no partner’ to the two items concerning the partner relationship
**Secondary Outcomes**		
Credibility of analogue therapy rationales	CEQ [47]	• Assesses the credibility of the rationales and procedures of the intervention and the optimal CAU control group • 4 items; 9-point Likert scale • Subscales: credibility; 1 single item (expectancy) • Cronbach’s alpha: credibility subscale: 0.81 ≤ α ≤ 0.86 [48]
Generic health-related quality of life related to gynaecological cancer	EORTC QLQ-C30 [41]	• Assesses QoL of cancer patients • 30 items; 4- and 7-point Likert scales • Subscales: 5 function subscales: physical; role; emotional; cognitive; social and 3 symptom subscales: fatigue; nausea/vomiting; pain. Single items: dyspnoea; sleep disturbance; appetite loss; constipation; diarrhoea; financial impact. One global QoL scale • Subscale scores: 0-100. Higher score indicates higher level of functioning (for the function subscales) and greater degree of symptoms (for symptom subscales and/or single items) • Time frame: past week • Cronbach’s alpha: subscales: 0.54 ≤ α ≤ 0.86 [41]
Vaginal symptoms and body image concerns	EORTC QLQ-CX24 [37]	• Assesses disease-specific and treatment-specific aspects of QoL in patients with cervical cancer • 24 items; 4-point Likert scale • Subscales: symptom experience; body image; sexual/vaginal functioning. Single-items: lymphedema; peripheral neuropathy; menopausal symptoms; sexual worry; sexual activity; sexual enjoyment • Subscale score: 0-100. Higher score indicates better level of functioning (for items regarding sexual activity and sexual enjoyment) and higher level of symptoms (for all other items and scales) • Time frame: past week (for the subscales and single-items lymphedema, peripheral neuropathy and menopausal symptoms); past 4 weeks (for the single-items sexual worry, sexual activity and sexual enjoyment) • Cronbach’s alpha: subscales: 0.72 ≤ α ≤ 0.87 [37]
Urological and gastrointestinal symptoms and sexual interest	EORTC QLQ-EN24 [42]	• Assesses urological and gastrointestinal symptoms, and sexual functioning • 10 items^#^; 4-point Likert scale • Subscales: urological symptoms; gastrointestinal symptoms. Single-item: sexual interest • Subscale score: 0-100. Higher score indicates higher level of urological and gastrointestinal symptoms and higher sexual interest • Time frame: past week • Cronbach’s alpha: subscales: 0.74 ≤ α ≤ 0.75 [42]
Quality of life	EQ-5D-5 L [49, 50]]	• Assesses (general) health • 5 items; 5-point Likert scale & Visual Analogue Scale (VAS) • 5 dimensions: mobility; self-care; usual activities; pain/discomfort; anxiety/depression. One VAS for general health • Time frame: today
Sexual distress	FSDS [39, 45]	• Assesses distress related to sexual dysfunction • 12 items; 5-point Likert scale • Total score: 0-48. Higher score indicates higher level of sexual distress • Time frame: past 30 days • Cronbach’s alpha: 0.86 ≤ α ≤ 0.94 [39]
Fear of coital and non-coital sexual activity	FSQ [38]	• Assesses aspects of fear of sexuality • 8 items; 5-point Likert scale • Subscales: fear of non-penetration sexual activity; fear of coitus/vaginal penetration • Subscale scores: fear of non-coital sexual activity 0-20; fear of coitus 0-12. Higher score indicates higher fear • Cronbach’s alpha: 0.82 ≤ α ≤ 0.86 [38]
Psychological distress	HADS [43, 51]]	• Assesses psychological distress • 14 items; 4 point Likert scale • Subscales: depression (HADS-D); anxiety (HADS-A) • Total score: 0-42/Subscale scores: 0-21. Higher score indicates more psychological distress • Time frame: past week • Cronbach’s alpha: HADS-D: 0.67 ≤ α ≤ 0.90; HADS-A: 0.68 ≤ α ≤ 0.93 [52]
Gynaecological cancer treatment related distress	IES [REF Brom, 1985; [40, 53]	• Assesses current treatment related distress • 15 items; 4-point scale • Subscales: intrusion; avoidance • Total score: 0-75/Subscale scores: intrusion 0-35; avoidance 0-40. Higher score indicates: higher tendency to be triggered by stimuli associated with the traumatic event(s) (for items regarding intrusion); higher tendency to avoid situations that are reminders of the treatment (for items regarding avoidance) • Time frame: past week • Cronbach’s alpha: total score: 0.87 ≤ α ≤ 0.96; intrusion subscale: 0.85 ≤ α ≤ 0.95; avoidance subscale: 0.77 ≤ α ≤ 0.91 [53]
Relationship dissatisfaction	MMQ Marital scale [44, 54]	• Assesses marital dissatisfaction • 10 items; 9-point Likert scale (range 0-8) • Total scale-score: 0-80. Higher score indicates higher marital dissatisfaction • Time frame: past 2 weeks • Cronbach’s alpha in non-distressed group: 0.87 ≤ α ≤ 0.88 [54]

*The score is calculated based on weighted items

^#^Due to the overlap between 4 items from the QLQ-EN24 and QLQ-CX24, we only included the remaining 6 items

CAU = Care as usual; CEQ = Credibility/Expectancy Questionnaire; EORTC QLQ-C30 = European Organization for Research and Treatment of Cancer Quality of Life Questionnaire-Core 30; EORTC QLQ-CX24 = European Organization for Research and Treatment of Cancer Quality of Life Questionnaire-Gynaecological Cancer Module; EORTC QLQ-EN24 = European Organization for Research and Treatment of Cancer Quality of Life Questionnaire-Endometrial Cancer Module; EQ-5D-5 L = EuroQol 5D-5 L; FSDS = Female Sexual Distress Scale; FSFI = Female Sexual Function Index; FSQ = Fear of Sexuality Questionnaire; HADS = Hospital Anxiety and Depression Scale; IES = Impact of Event Scale; MMQ = Maudsley Marital Questionnaire; QoL = Quality of Life

Additionally, the secondary outcome measures include a 4-item questionnaire for women treated with EBRT+BT regarding the frequency of dilator use: (1) ‘How often have you used the dilator in the past month?’, (2) ‘Which size(s) dilator(s) did you use?’, (3) ‘How often did you have other kinds of penetration (including penile penetration) in the past month?’, and (4) ‘How often did you use petroleum jelly (Vaseline) tampons?’.

Furthermore, the following vaginal symptoms are assessed by standardized clinical examination for all participants: dryness, shortening/tightening, mucositis, discharge, blood loss, fibrosis, atrophy, pain, length (in millimetre) and dyspareunia. Vaginal symptoms will be recorded using the Common Terminology Criteria Sexual rehabilitation after RT for gynaecological cancers for Adverse Events (CTCAE), version 4.037.

#### Cost-effectiveness

A cost-effectiveness analysis (CEA) will be conducted in which the costs of the intervention will be related to shifts in other health care costs and the impact on patient outcome. A cost-price analysis will be performed for the nurse-led sexual rehabilitation intervention (including training, counselling hours and materials). Other healthcare use will be limited to sexuality-related health care utilization (including gynaecologist, radiation oncologist, general practitioner, psychologist and sexologist) and medication use, estimated from patient reports and valued using standard prices.

Estimated costs will be related to the impact on the number of women with sexual improvement after 12 months (costs-per-improved-patient, defined as a Reliable Change Index (RCI) [55] >1.96 on the FSFI [36] total score) and to the impact on quality-adjusted life years (cost-per-QALYs). In the primary analysis, consistent with Dutch guidelines, QALYs will be calculated using the Dutch tariff for the 5-level EuroQol-5D (EQ-5D-5 L) [49, 50, 56]. As a secondary analysis, QALYs will also be estimated using the EuroQol visual analogue scale (EQ-VAS, with power transformation) as predicted by a mapping from the FSFI to the EQ-VAS. This secondary analysis is included because the Dutch tariff for the EQ-5D-5 L does not explicitly value sexuality and mapping from the FSFI will make the approach more sensitive to change.

#### Other study parameters

In addition to treatment- and patient characteristics, treatment credibility and expectancy for improvement will be assessed using the 4-item Credibility and Expectancy Questionnaire (CEQ) [47]. These parameters will be measured at T3. Additionally, at T2-T5 all women will be requested to report the use of any counselling or therapy in the course of their rehabilitation period. Furthermore, the type (face-to-face or by telephone) and the duration of the session, and modules used during the session are documented by the oncology nurses in CRFs. Finally, for each participant, the date of the completion of study participation and the reason for ending study participation are registered in web-based CRFs.

### Statistical methods

#### Power and sample size calculation

The FSFI [36] is the primary outcome measure on which sample size calculations are based. With a total sample of 128 women (64 per group), and under the assumption of no interaction, the study will have a 80% power to detect a 0.5 standard deviation difference (Cohen’s effect size [57]) for the main effects of the sexual rehabilitation intervention, with the p-value set at 0.05 (two-sided test). Based on our pilot study we expect an attrition rate of 40% (i.e., women who discontinue participation in the study due to somatic reasons such as cancer recurrence) [32]. Consequently, we have to include at least 107 women in both study groups ultimately resulting in an intended sample size of 220 women [57].

### Statistical analysis

T-tests or appropriate non-parametric statistics for independent samples will be used to evaluate the comparability of the intervention and control group at baseline in terms of sociodemographic and clinical characteristics. If, despite the stratified randomization procedure, the groups are not comparable on one or more background variables, those variables will be employed routinely as covariates in subsequent analyses.

Questionnaire scores will be calculated according to published scoring algorithms. Differences in changes in the primary outcome measure and secondary outcome measures between groups (intervention vs. control group) over time (T1-T5) will be evaluated using multilevel models and are based on an intention-to-treat approach. Effect sizes will be calculated using standard statistical procedures.

Furthermore, multilevel models will be used to investigate if improvement in sexual functioning in the intervention group is moderated by treatment/cancer characteristics and patient characteristics or mediated by e.g. sexual symptoms and vaginal symptoms or frequency of dilator use. Cost-effectiveness will be analysed using net benefit analysis, with multiple imputation to account for missing data.

Per protocol analyses will also be carried out (as a secondary analysis), comparing women who meet minimal compliance levels with the intervention with the control group. We will use correlation analyses to examine the relationship between degree of intervention adherence and intervention effect.

## Discussion

Sexual problems, such as dyspareunia, vaginal dryness and a decrease of sexual satisfaction and desire, are frequently reported by GC survivors after treatment with RT, and occur in particular after combined EBRT+BT. Previous studies have shown that psychosexual support after treatment for GC should include cognitive behavioural interventions to increase dilator compliance and improve sexual functioning [2, 15, 20–26]. Furthermore, including the patient’s partner, if available, is preferred [26]. Therefore, we developed a nurse-led sexual rehabilitation intervention, including these key components, to support sexual improvement and vaginal dilator use after RT. This RCT will provide evidence about the efficacy of this nurse-led sexual rehabilitation intervention in terms of sexual functioning as measured by the FSFI [36], as well as evidence on other sexual outcome measures, compliance with vaginal dilation and the cost-effectiveness of the rehabilitation intervention. We expect that women who received the intervention will report a statistically significantly greater improvement in sexual functioning and – for women who receive EBRT+BT – higher compliance with dilator use, from baseline to 12 months post-RT than women who receive optimal CAU. If proven effective, the rehabilitation intervention will be a valuable addition to the care offered to GC survivors and will contribute to improved quality of life after GC.

The SPARC study has several notable strengths, including the randomized trial design, the multicentre nature (with participation of all Dutch GC centres), the comparison of the intervention group with a control group, the use of a clear treatment protocol and training protocol, the use of intention-to-treat analyses and the long-term follow-up assessments of outcomes. This trial also has several limitations. First, even though the FSFI is one of the most widely used questionnaires to measure sexual functioning among female cancer survivors, it produces biased results for women who have not been sexually active in the past month [36, 58]. The majority of the questions (15 out of 19) include a response option of ‘No sexual activity’ or ‘Did not attempt intercourse’, scored as zero. This is problematic because lower scores indicate more severe dysfunction whereas not engaging in sexual activity during four weeks does not necessarily imply sexual dysfunction. Sexual inactivity could have multiple reasons, such as the absence of a partner. As our study sample consists of partnered as well as unpartnered women, we chose to randomize participants stratified by having a partner. Second, despite the proposed benefits of regular dilator use (i.e., preventing or minimising vaginal stenosis), unequivocal evidence for its effectiveness in reducing vaginal complaints and better sexual functioning is still limited. The consequences of stenosis remain individually determined, with some women unaffected by significant vaginal complaints, while others experience long-lasting sexual problems [11]. However, because dilator use aims to prevent or minimise stenosis, we expect that this could positively affect vaginal complaints and sexual functioning indirectly. Therefore, we will include dilator use as a mediator instead of an outcome measure in the statistical analyses. Third, because RT-induced sexual problems develop soon after treatment, we start evaluating GC survivors early in the recovery phase and continue until 12 months post-RT. A longitudinal study that analysed functioning and symptom scores for quality of life of cervical cancer patients who underwent EBRT+BT demonstrated that RT-induced sexual problems increased to a clinically relevant degree in the first three months, after which it stayed elevated, even after 12 months [59]. This study did not include a sexual rehabilitation intervention. By offering a nurse-led sexual rehabilitation intervention we hope to find significant greater improvement in sexual functioning between the intervention- and optimal CAU control group at 12 months follow-up. However, it is possible that sexual functioning among GC survivors might improve further after 12 months post-RT. Therefore, we intend to plan an additional long-term follow-up measure at 24 months post-RT. Fourth, the possibility of contamination of our optimal CAU control group exists. In the past few years, the Dutch CAU regarding sexual problems after RT improved and was aligned nationwide, possibly as a consequence of the findings of our pilot study [32]. This resulted in fewer differences between the participating centres in the current trial, as all participating women are offered an information session, information booklet and a dilator set (if applicable) post-RT free of charge. In the current study, this improved CAU, combined with the possibility that the specialized trained nurses also come into contact with the control group, may result in the control group receiving better post-RT psychosexual care than intended - as the nurses might find it difficult not to use the additional trained skills to help these patients. This well-known problem of contamination within individually randomized intervention studies could be avoided by cluster randomization (i.e., on centre level instead of patient level). However, this method also introduces other potential threats to internal validity, as the number of centres in our study is limited and only a part of the centres could be randomized (n = 8, as a consequence of the training that was already completed in two centres for the pilot study) [32]. Because of the specific variation in the patient population, RT treatment procedures and follow-up procedures across centres, we decided to randomize on patient level, with the risk of contamination. During their training, the nurses received clear instructions on the procedure to be followed in both study groups and about the contamination risks.

The importance of the availability of a sexual rehabilitation intervention is evident from the British [60], Australian [61] and Dutch guidelines [62] which state that more attention has to be paid to sexual functioning after RT for GC. To our knowledge, this is the first RCT evaluating the (cost-)effectiveness of a nurse-led sexual rehabilitation intervention in improving sexual functioning and dilator use compliance of GC patients after RT. If proven to be effective, the intervention will be a valuable addition to GC survivors’ standard care. It will ultimately improve the quality of life of patients (and their partners). The intervention can be implemented nationwide directly after study completion, as all end-users were involved in the preparatory studies [17, 18, 32] and nurses in all Dutch GC centres are trained in the treatment protocol. Implementation is further enhanced by the relatively low costs of personnel and materials. In addition, if successful, the intervention could be extrapolated to women with other types of pelvic cancer (e.g., rectal cancer, anal cancer and bladder cancer).

## Data Availability

All (Dutch) materials, such as information folders, will be available by the end of current study and publication of the results. Data containing potentially identifying or sensitive patient information are restricted according to European law (General Data Protection Regulation (GDPR)). The datasets generated and/or analysed during the current study are not available in a public repository, but are available upon reasonable request via MtK.

## Abbreviations

BT: Brachytherapy
CAU: care as usual
CEA: cost-effectiveness analysis
CEQ: Credibility and Expectancy Questionnaire
CRF: Case Report Form
CTCAE: Common Terminology Criteria Sexual rehabilitation after RT for gynaecological cancers for Adverse Events
EBRT: external beam radiotherapy
EORTC QLQ-C30: European Organization for Research and Treatment of Cancer Quality of Life Questionnaire Core 30
EORTC QLQ-CX24: European Organization for Research and Treatment of Cancer Quality of Life Questionnaire-Gynaecological Cancer Module
EORTC QLQ-EN24: European Organization for Research and Treatment of Cancer Quality of Life Questionnaire-Endometrial Cancer Module
EQ-5D-5L: 5-level EuroQol-5D
EQ-VAS: EuroQol visual analogue scale
FIGO: Fédération Internationale de Gynécologie et d’Obstétrique
FSDS: Female Sexual Distress Scale
FSFI: Female Sexual Function Index
FSQ: Fear of Sexuality Questionnaire
GC: gynaecological cancer
HADS: Hospital Anxiety and Depression Scale
IES: Impact of Event Scale
min: minutes
MMQ: Maudsley Martial Questionnaire
PROM: patient-reported outcome measure
QALYs: quality-adjusted life years
RCI: Reliable Change Index
RCT: randomized controlled trial
RT: radiotherapy
SPARC: Sexual rehabilitation Programme After Radiotherapy for gynaecological Cancer
T1: baseline
T2: one month post-radiotherapy
T3: three months post-radiotherapy
T4: six months post-radiotherapy
T5: twelve months post-radiotherapy
WHO: World Health Organisation
